# Botanical Analysis of the Baroque Art on the Eastern Adriatic Coast, South Croatia

**DOI:** 10.3390/plants12112080

**Published:** 2023-05-23

**Authors:** Nenad Jasprica, Vinicije B. Lupis, Katija Dolina

**Affiliations:** 1 Institute for Marine and Coastal Research, University of Dubrovnik, Kneza Damjana Jude 12, HR 20000 Dubrovnik, Croatia; nenad.jasprica@unidu.hr; 2 Institute of Social Sciences Ivo Pilar, Regional Center in Dubrovnik, HR 20000 Dubrovnik, Croatia; vinicije.lupis@pilar.hr

**Keywords:** art, Baroque, floral elements, NE Mediterranean, Pelješac peninsula, sacral heritage

## Abstract

The analysis of plants featured in Baroque artworks on the eastern Adriatic coast has not previously been the subject of an in-depth study. The study of plant iconography in Baroque sacred artworks, which are mostly paintings, was carried out in eight churches and monasteries on the Pelješac peninsula in southern Croatia. Taxonomic interpretation of the painted flora on 15 artworks led to the identification of 23 different plant taxa (species or genera) belonging to 17 families. One additional plant was identified only by family taxonomic rank. The number of plants was relatively high, and most species were considered non-native (71%, “exotic” flora) phanerophytes. In terms of geographic origin, the Palaearctic region (Eurasia) and the American continent were identified as the main areas of plant origin. *Lilium candidum*, *Acanthus mollis*, and *Chrysanthemum* cf. *morifolium*, were the most common species. We think that the plants were selected for decorative and aesthetic reasons, as well as for their symbolic significance.

## 1. Introduction

Plant science research is accelerating at a rapid pace in Croatia [1]. New technologies and expanding infrastructure development opened the door to cutting-edge research on a large scale. Despite this noteworthy growth, access to old artworks is not evenly distributed across the country. In fact, we identified a research gap in the plant study of artworks in the eastern Adriatic region. Actually, there is no place or artwork that was previously studied scientifically in such detail. This study may be classified as an inter-disciplinary approach designed to enable the interpretation of botanical species and facilitate a better understanding of the context of Baroque art in the area of interest. Besides the scientific problem of accurately defining the typology of the plants represented, an attempt should be made toward decoding the message underlying the decoration. In general, botanical analysis of artworks considers their physical–natural, historical, and ideological aspects as they change throughout history. This perspective can contribute to a more objective enhancement of this complex cultural heritage in which nature and culture are intertwined.

The importance of the study of plant iconography and, in general, the floristic richness of the artworks in the Mediterranean was highlighted in the last decade. The floristic richness in Roman iconography and the plants carved in the fountain (Rome, mid-17th century) were analyzed on the basis of iconographic and historical documents [2,3]. Hosseini and Caneva [4] emphasized the lack of a general methodological approach for the simultaneous evaluation of historical, structural (i.e., composition), and botanical features, as well as for revalorization the natural components of the lost gardens from antiquity. Images of date palms (*Phoenix dactylifera* L.) on coins were analyzed from agricultural, botanical, and geographic perspectives, particularly with respect to their relationship with the climatic conditions that were favorable for their cultivation [5].

Baroque is a style of architecture, painting, sculpture, and other arts that followed Renaissance and Mannerist art and preceded Rococo (often referred to as “late Baroque”) and Neoclassicism [6]. The Baroque era began in Rome, Italy, in the early 17th century and then spread rapidly to France, Northern Italy, Western Europe (Spain, Portugal), and other countries. In general, the Baroque style developed in different regions at different times, but was established throughout Europe by 1620 [7]. In Europe, the Baroque style influenced all aspects of the visual and performing arts in the 17th and 18th centuries [8]. In some areas (e.g., the Iberian Peninsula), it continued along with new styles in the first decade of the 19th century.

The Baroque style introduced a variety of thematic innovations to the visual arts, such as vedute, still life, battle scenes, magnificent landscapes, plants, fruits, etc. [9]. Many painters used a very realistic style. There was much attention to detail, albeit not with the scientific precision and detail of Renaissance art (1400–1540). In contrast to the Renaissance, the Baroque landscape did not focus on human figures, while nature was given prominence in the composition. However, representations of plants and, more generally, of natural elements were not merely decorative or chosen for aesthetic reasons, but often pursued a specific symbolic purpose [10]. We agree with Caneva [11] and Caneva et al. [12], who argue that people in the past were able to understand these symbolic meanings thanks to their deep connection with and understanding of their environment.

At that time, the entire region along the Dalmatian coast (the eastern Adriatic), with the exception of the city-state Republic of Dubrovnik (or Republic of Ragusa), belonged to the Republic of Venice. The entire area, from Istria in the north to the Bay of Kotor in the south, was predominantly under the influence of the Venetian school of art, while artists from central and southern Italy, especially Naples, left visible traces in Dubrovnik and its surroundings [13,14].

The Baroque period on the Pelješac peninsula was an extremely intense period of building and art acquisition, because it was the time when the rich class of sailors and shipowners, especially those located in the western part of the peninsula, started building more sacral monuments [15]. Among the sacred works of art, altarpieces with very popular motifs dominated in this period. These motifs are mostly associated with the cult and veneration of the Mother of God and various saints, from martyrs from early Christianity to ‘new’ local patron saints of Croatian coastal communities and dioceses (St. Anthony of Padua, St. Blasius, St. Anne, etc.). In addition, floral patterns are found on these paintings and architectural elements in Baroque churches and monasteries in the region [16,17].

The objectives of our study were as follows: (i) to analyse the presence of floral elements in Baroque sacred art on the Pelješac peninsula; (ii) to determine the relationship between the local flora and the flora recognisable on the artworks; and (iii) to contribute to a better understanding of the relationship between man and the environment in this area.

## 2. Materials and Methods

### 2.1. Study Area

**Figure 1:**
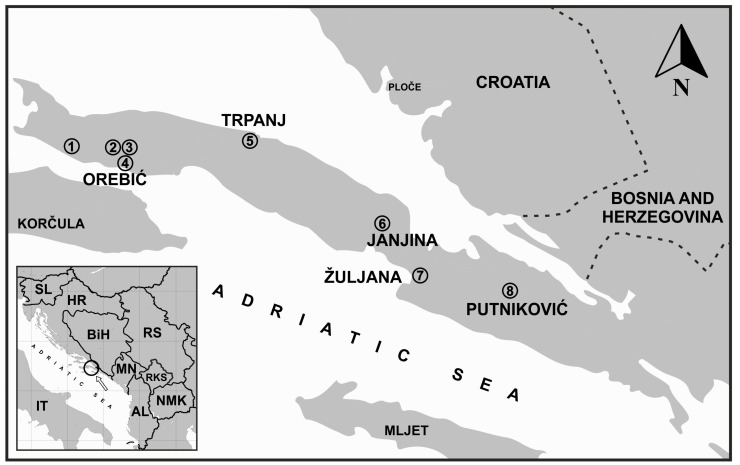
Map of Pelješac peninsula and its location on southeastern Adriatic coast (SE Europe). Numbers indicate location of churches and monasteries where artworks were examined: 1—St. Anne Chapel, Žukovac, near village of Kućište; 2—Franciscan Monastery and Church of the Great Lady, Podgorje; 3—Church of Our Lady of Carmel, village of Carmel, Podgorje; 4—Church of Christian’s helpers, Orebić; 5—St. Anthony of Padua Church, Trpanj; 6—St. Blasius Church, Janjina; 7—St. Martin Church, Žuljana; 8—Church of Our Lady of the Rosary, Tomislavovac, near village of Putniković (for a detailed description see Section 2.2). Abbreviations: IT—Italia, SL—Slovenia, HR—Croatia, BiH—Bosnia and Herzegovina, MN—Montenegro, RS—Serbia, RKS—Kosovo, AL—Albania, NMK—North Macedonia. The circle on the map in the lower left corner indicates the research area in the SE European context.

The Pelješac peninsula (area 355 km^2^, max. altitude 961 m a.s.l.) is located on the eastern Adriatic coast in southern Croatia (Figure 1). Archaeological findings from the western part of the peninsula indicate continuous human settlement for several millennia [18]. However, the earliest known historical records of Pelješac date back to ancient Greece. After the Illyrian Wars (220 to 219 BC), the area became part of the Roman province of Dalmatia. Human activities have affected the environment for thousands of years [19,20]. In the mid-17th century, Pelješac was located quite far from the nearest major urban center (Dubrovnik) and had about 8000 inhabitants [21]. The population fluctuated from the 15th to the 17th century due to the immigration of Christian refugees from Bosnia and Herzegovina, epidemics, the Cretan War (1645–1669), the earthquake of 1667, and emigration. In general, the majority of the population was poor and engaged in fishing and agriculture, while shipping and international maritime trade increased in the 16th and 17th centuries.

Phytogeographically, the peninsula belongs to the Mediterranean Region, the Eastern Mediterranean Subregion, Adriatic Province, and the Epiro-Dalmatian Sector (*sensu* [22]). It is predominantly composed of carbonate rocks. The climate in this area is Mediterranean with mild, humid, and rainy winters and dry and hot summers (*Csa* subtype of Mediterranean climate, *sensu*) [23,24]. This climate enables the development of eu-Mediterranean vegetation dominated by evergreen shrubs and sclerophyllous trees (maquis), with the most important tree species being the holm oak (*Quercus ilex* L.). Today, the Pelješac peninsula is one of the Important Plant Areas (IPAs) in Croatia and has a high structural diversity of vegetation [25,26]). On the peninsula, there are sites rich in endemic flora [27], while the larger part of the peninsula is covered by the NATURA 2000 network of protected areas in Croatia [28,29].

### 2.2. Methods

The study of the plant iconography of the artworks was carried out in eight churches and monasteries on the Pelješac peninsula (Figure 1). A total of 15 artworks were analysed.

The criterion for the selection of the artworks was based on territorial and representative principles. For this purpose, we first studied the documents of the Museum Documentation Center (MDC), i.e., the Register of Museums, Galleries, and Collections in the Republic of Croatia, which contains relevant information about the collections and their artworks. However, a significant part of the cultural heritage is owned by religious communities, which keep the artworks in their collections and treasuries. Although not all collections owned by religious communities were included in the Register, it is clear that there are many more collections than are officially recorded [16,17]. In addition, we had only partial insight into the list of artworks in Catholic parishes on the peninsula, as in many cases such lists are missing. Therefore, we visited all available churches that we knew contained artwork and attempted to cover the entire peninsula area. Access to some artworks in churches and monasteries was not possible for various reasons (restoration, loan to other parties, etc.). Our main focus was on artworks with floral motifs located on the Pelješac peninsula that date from the Baroque period, whose creators were local artists (e.g., Filippo Naldi) who skillfully contributed to the development of new motifs in artistic expression and decorated Baroque interiors with plant motifs. The artworks considered in this study are an exclusive example of folk Baroque on the Pelješac peninsula, which emerged during the period of the Dubrovnik Republic. All the artworks considered come from places located on the peninsula, most of which correspond to settlements where a newly enriched maritime folk class with its own cultural needs began to develop.

The artworks are listed in Table 1 (see also Table 2).

The species presented were identified on the basis of the most diagnostic morphological aspects, such as the general form of the plant (habit), typology, shape, size, and color of the flowers and fruits, if present, and the morphology and arrangement of the leaves. The correct number of single parts was more or less easy to identify in real individuals (specimens), though this was not always possible in painted or engraved elements. This identification became even more difficult when time-related damage was added to the sometimes-poor accuracy of the painter in depicting plants. Therefore, in the absence of precise diagnostic elements, an assignment based on considerations related to habitat and probable abundance in adjacent natural contexts was proposed (see Caneva and Bohuny [30], Caneva et al. [3]). When interpretation was too doubtful or ambiguous, identification was restricted to a general (higher taxonomic) level.

Various floras were used to determine the plants according to their diagnostic elements and their ecological and biogeographical aspects (for details, see Jasprica and Milović [31], Milović et al. [32], and Jasprica et al. [33], as well as references therein). Matthioli [34,35] was also consulted for iconographic analysis. The nomenclature of plant taxa follows the Plants of the World Online database [36]. The floristic list below (Table 2) includes the following aspects: the updated scientific name, the common name in English and Croatian (in parentheses), the structure expressed by the biological forms, and the chorology (geographical origin). Plants in the floristic list are given in alphabetical order. The frequency of their occurrence in the paintings and the elements that led to their identification are also indicated. Croatian common names, mainly used on the Pelješac peninsula, were identified using the Nomenclator botanicus Croaticus [37] and the Flora Croatica Database [1].

## 3. Results

**Figure 2:**
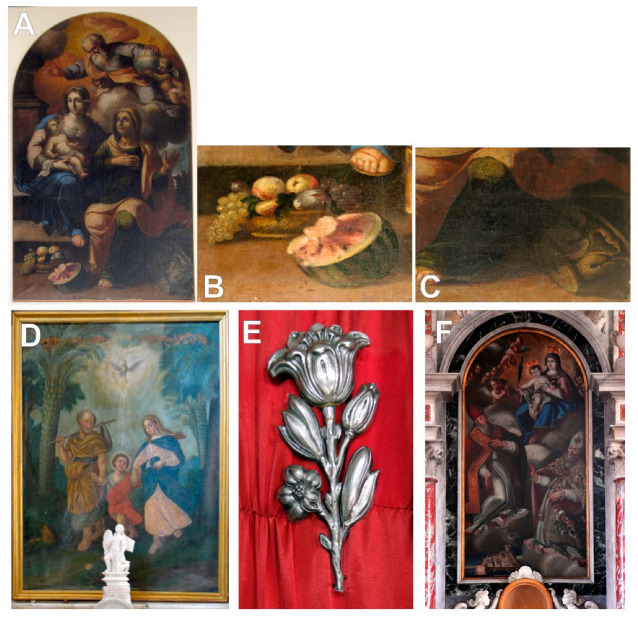
(**A**)—St. Anne and Our Lady with Jesus Christ and Our Father in Chapel of St. Anne in Kućište; (**B**,**C**)—details from lower left and right parts of paintings; (**D**)—The Escape to Egypt, and (**E**)—Health-related Votive Tablet in Church of Christian’s helpers in Orebić; (**F**)—The Virgin with Child, St. Blasius, and St. Nicholas in parish church of St. Blasius in Janjina.

**Figure 3:**
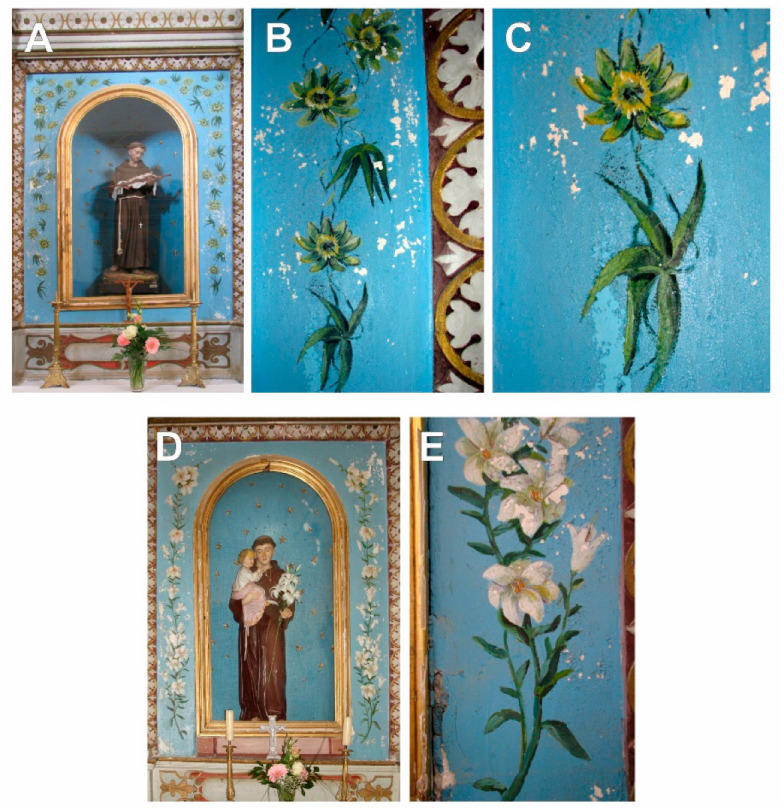
The altars of St. Francis of Assisi (**A**) with details on *Passiflora caerulea* L. (**B**,**C**); St. Anthony of Padua (**D**) and details on *Lilium candidum* L. (**E**) in Franciscan Monastery and Church of the Great Lady in Podgorje.

**Figure 4:**
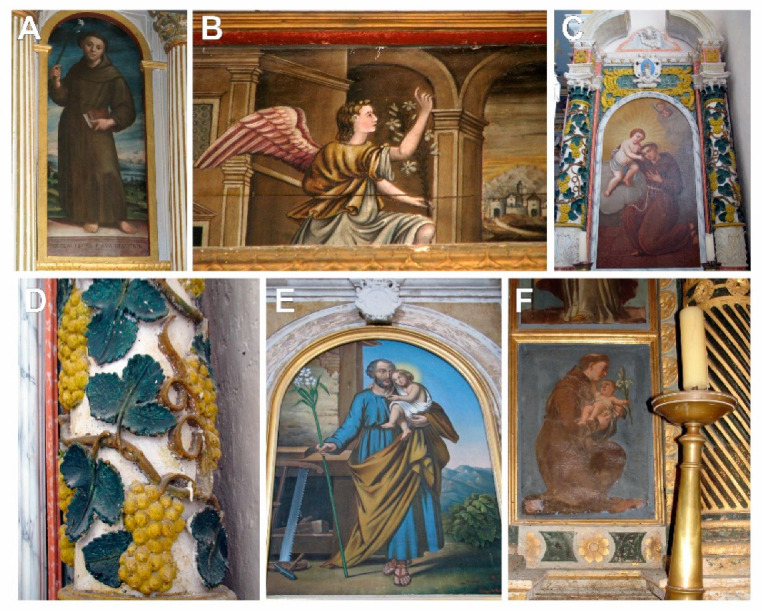
(**A**,**C**,**D**,**F**)—The altars of St. Anthony of Padua; (**B**)—The Archangel Gabriel, (**E**)—St. Joseph in Podgorje.

**Figure 5:**
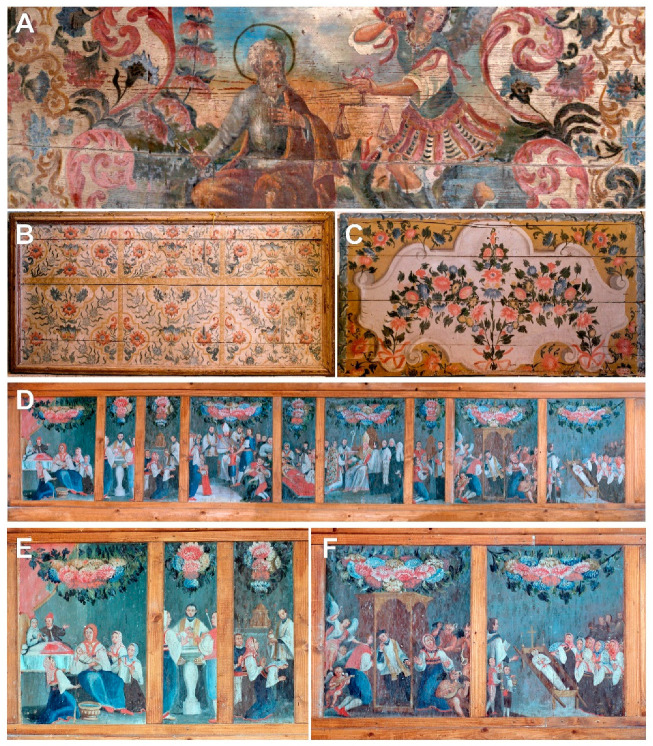
(**A**)—Antependium in Church of St. Anthony of Padua in Trpanj; (**B**,**C**)—Antependia in Church of St. Martin in Žuljana; (**D**)—Polyptych Birth and Death with the Seven Holy Sacraments on wooden fence by choir in Church of Our Lady of the Rosary in Putniković; (**E**,**F**)—Details of first and final parts of polyptych.

The taxonomic interpretation of the painted flora led to the identification of 23 different plant taxa (species or genera) belonging to 17 families. One additional plant was identified only via family taxonomic rank (Table 2, Figure 2, Figure 3, Figure 4 and Figure 5).

The most common plants (occurring in at least four artworks) were *Lilium candidum*, *Acanthus mollis*, and *Chrysanthemum* cf. *morifolium*.

*Prunus* and *Rosaceae* were the most represented genera and families, respectively (Table 2). The analysis of plant life forms showed that the artworks were dominated by phanerophytes (54%), followed by therophytes (21%).

Most species were non-native (71%, also referred to as “exotic” flora) and originated mainly from Asia. They were largely cultivated for their nutritional value (fruits, vegetables) and as ornamentals. Among the native taxa, Mediterranean floral element (i.e., *Centaurea cyanus*, taxa from the Orchidaceae family), which were mostly circum-Mediterranean plants, predominate. Although some plants (*Acanthus mollis*, *Vitis vinifera*) were signed as originating from outside the Mediterranean region, they have been cultivated since ancient times and, thus, have become an integral part of the local wild flora in the Mediterranean region.

**Table 2:** Identification and distribution of floristic elements painted on artworks of Pelješac peninsula. Corresponding family and Croatian name of genera or species are given in parentheses. Abbreviations, life-form: G—geophytes, H—hemicryptophytes, P—phanerophytes, T—therophytes; chorotype (biogeographic element): EA—Eurasian, AS—Asian, AF—African, Medit.—Mediterranean, AM—Americas, EU—European, Cos—Cosmopolitan. It is also indicated whether plant grows naturally (native) in Croatia or is non-native. Codes of artworks in which plant appears are given in Table 1 in Section 2.2.

Proposed Identification		Painted Part of the Plant	Life-Form	Chorotype, Native or Non-Native	Frequency of Occurrence (Code of Artworks in Which the Plant Appears)
**Scientific Name**	**Common Name**				
*Acanthus mollis* L. (Acanthaceae)	Common bear’s breech (meki primog)	Leaves	H	AF, Medit., non-native	4 (1, 7, 11, 12)
*Campsis radicans* (L.) Bureau (Bignoniaceae)	trumpet vine (tekoma)	Flowers, inflorescence	P, liana	AM, non-native	1 (15)
*Centaurea cyanus* L. (Asteraceae)	Cornflower, bachelor’s button (različak, zečina)	Flowers, inflorescence	T	Medit., native	1 (11)
*Chrysanthemum* cf. *morifolium* (Ramat.) Hemsl. [incl. *C*. *indicum*] (Asteraceae)	Florist’s daisy, garden mum (krizantema)	Flowers, inflorescence	T	AS, non-native	4 (9, 13–15)
*Citrullus lanatus* (Thunb.) Matsum. and Nakai (Cucurbitaceae)	Watermelon (sađena lubenica)	Fruit	T	AF, non-native	1 (1)
*Dianthus* cf. *caryophyllus* L. (Caryophyllaceae)	Carnation (pitomi klinčić)	Flowers	H	EU, native	1 (11)
*Hedera helix* L. (Araliaceae)	Common ivy (obični bršljan)	Leaves	P, liana	EU, native	1 (9)
*Hydrangea macrophylla* (Thunb.) Ser. (Hydrangeaceae)	bigleaf hydrangea (velikolistna hortenzija)	Flowers, inflorescence	P	AS, non-native	1 (15)
*Justicia carnea* Lindl. (Acanthaceae)	Brazilian plume flower, jacobinia (dubrovački gospar)	Flowers, inflorescence	P	AM, non-native	1 (11)
*Knautia*/*Dipsacus* (Caprifoliaceae)	Widow flower/teasel (prženica/češljugovina)	Flowers, inflorescence	H	EA/EA, AF, native	1 (13)
*Leonotis leonurus* (L.) R.Br. (Lamiaceae)	Lion’s tail, wild dagga (lavlji rep)	Flowers, inflorescence	P	AF, non-native	1 (11)
*Lilium candidum* L. (Liliaceae)	White lily, Madonna lily (bijeli ljiljan)	Flower, inflorescence	G	EA, native	7 (3, 4, 6–10)
*Malus domestica* (Suckow) Borkh. (Rosaceae)	Apple (obična jabuka)	Fruit	P	AS, non-native	1 (1)
Orchidaceae	Orchid (orhideje, kačunovice)	Flower shape and structure	G	Cos, native	2 (2, 3)
*Paeonia* sp. (Paeoniaceae)	Peony (božur)	Flowers	G	EA, AF, AM, non-native	1 (13)
*Papaver rhoeas* L. (Papaveraceae)	Common poppy (divlji mak)	Flowers	T	EA, native	2 (11, 12)
*Passiflora caerulea* L. (Passifloraceae)	Blue passionflower (krunica gospodinova)	Flowers, leaves	P, liana	AM, non-native	1 (2)
*Phoenix dactylifera* L. (Arecaceae)	Date palm (obični datuljevac)	Entire plant	P	AS, non-native	1 (9)
*Prunus domestica* L. (Rosaceae)	European plum (obična šljiva)	Fruit	P	EA (Türkiye), non-native	1 (1)
*Prunus persica* (L.) Batsch (Rosaceae)	Peach (breskva)	Fruit	P	AS, non-native	1 (1)
*Pyrus communis* L. (Rosaceae)	Common pear (obična kruška)	Fruit	P	EA, non-native	1 (1)
*Rosa* sp. (Rosaceae)	Rose (ruža)	Flowers	P	EA, AM, AF, non-native	2 (12, 13)
*Solanum melongena* L. (Solanaceae)	Eggplant (balančana)	Fruit	T	AS, non-native	1 (1)
*Vitis vinifera* L. (Vitaceae)	Grapevine (vinova loza)	Leaves, fruit	P, liana	EA, non-native	2 (1, 5)

## 4. Discussion

In this study, the number of plants is relatively high, and most species were considered non-native (“exotic” flora) phanerophytes, most of which are cultivated for various purposes. The main areas of origin of the plants were identified as the Palaearctic and the Americas [38]. Despite the great diversity of plants, a repetitive trend in the occurrence of species can be seen. *Lilium candidum*, *Acanthus mollis* and *Chrysanthemum* cf. *morifolium*, followed by *Vitis vinifera*, *Papaver rhoeas*, orchids and various *Rosa* spp., were the most common species.

Both *A. mollis* and *V. vinifera* (grapevine) are widely used in paintings, mosaics, and classical sculptures [2,39]. In general, these species, including some others found in this study (e.g., *Phoenix dactylifera*), are very common in sacred artworks due to their strong association with mythological and religious symbolic meanings. The frequent occurrence of *A. mollis*, which is a wild species in northwest Africa and the Mediterranean region, is related to the idea of “rebirth” and its symbolism [40,41]. The morphological characteristics of the grapevine (i.e., the single fruit is round, fleshy, and bears more than one seed) evoke specific symbolic meanings associated with ideas of wealth, fertility, and prosperity. However, the primary symbolic meaning of the grapevine is actually associated with notions of life and vitality [10].

The historical data prove the centuries-old tradition of cultivation of different varieties of *V*. *vinifera* on the Pelješac peninsula, which has been persevered until today [42]. In the case of *A. mollis*, the interpretation of its presence in this area must include an analysis of the activities of the Republic of Dubrovnik when it was an important thalassocracy. In that period, especially in the late Baroque, the ships and emissaries of the Republic often returned to port with exotic plants that they had collected along the Mediterranean coast and beyond (for a review, see Đurasović [43].

In this study, *Lilium candidum* was the “attribute” of St. Anthony of Padua, as well as of St. Joseph and the Archangel Gabriel. The white lilies (also called “Madonna lilies”) bloomed at Easter, sprang from the staff of St. Joseph, and were carried by the Angel of the Annunciation [40,44]. To this day, St. Anthony is one of the most venerated and popular saints of the Catholic Church in Croatia, because his life was a constant struggle to face the ups and downs of life [45]. However, in church symbolism, the “lily of purity” is particularly suitable to represent the Virgin and adorn her altars [46], which was not the case in this study.

Although *Chrysanthemum* cf. *morifolium*, which was commercially introduced to Europe from China in the late 18th century, has been used as a medicinal, food, and ornamental plant for at least 2200 years [47], in our case, it has more cultural significance. In Croatian tradition, chrysanthemum is strongly associated with death [48]. Chrysanthemums can send a message of remembrance to the deceased, but they also convey to the living the family’s commitment to the memory of a deceased loved one; this cultural belief is also reported in other European countries [49]. However, Moore [50] noted that there are several inconsistencies in defining floral meanings across generations, i.e., from funerals, mourning, and condolences to homecomings and celebrations. Morphologically, *Chrysantemum* cf. *morifolium* has some similarities with *C*. *indicum*. Therefore, due to the very complex taxonomic implications, we included *C*. *indicum* in addition to *Chrysanthemum* cf. *morifolium* in the floristic list (Table 2).

Chrysanthemums and various *Rosa* spp. are common plant species on the wooden antependia and fence at the choir (see Figure 5), which was painted by Filippo Naldi (?–1783), who was from Florence, Italy, and belonged to the group of artists who influenced 18th century Dalmatian art [17,51]. His depictions of sacred artwork on the Dalmatian coast were rich in floral patterns, while the figures are enriched with representations of floral ornaments covering the clothing [14]. However, echoes of Venetian painting of the Seicento and Settecento can also be seen in his works [14,51,52].

Meagher [53] emphasises that the presence of fruits (e.g., *Solanum melongena*, *Citrullus lanatus*, etc.) reflects the influence of the current stream of profane still life painting in Southern Europe. In the present study, some plants (*Papaver rhoeas*, *Centaurea cyanus*, *Passiflora caerulea*, etc.) were found to be used in traditional or official medical practice in the eastern Adriatic islands [54]. Of all the islands, the longest list of medicinal plants used was recorded on the neighbouring island of Korčula, and most of these plants already appear in ancient and mediaeval herbal books [55]. For example, *P. rhoeas* is characterised by its sedative effect, and *C. cyanus* was mainly used to cure eye inflammations [56]. On the other hand, the presence of these two species could indicate more intensive agriculture in that period on the Pelješac peninsula. Nowadays, *C. cyanus*, which is a weed of cereal fields and olive groves, does not occur in this area, probably due to environmental changes, i.e., habitat loss or possible impact of management practices in olive groves (for a literature review, see *Flora Croatica Database* [1]). Finally, Pinke et al. [56] pointed out the deep cultural embeddedness of these charismatic arable weeds and their symbolic connotations related to human characters and feelings (patriotism, historical remembrance, virginity, loyalty, etc.).

The methodological limitations of the study must be emphasised. The highest number of plants was determined based on their flowers and fruits. The habit and the typical morphology of the leaves were used as good diagnostic elements for the herbaceous species. However, naturalistic descriptions of species are not rigorous, and their identification is often based on a few diagnostic elements, e.g., trees and shrubs are often identifiable based on their fruits. In the case of *Rosa* spp. and several other plants, the lack of details in habit or organs (e.g., flowers with petals, basal leaf rosette, etc.) made it impossible to identify them to species level. This problem was also emphasised by other studies, e.g., Caneva and Bohuny [30] and Caneva et al. [3]. In addition, the species represented a lack of seasonal consistency, in that some are species depicted as growing in springtime while some others are growing in autumn. For example, *A. mollis* has a specific phenology: it appears dead in summer but regrows after the first fall. *Vitis vinifera* is without leaves in winter and appears to be dead, but comes back to life in the growing season. The latter phenomenon is connected with the ideas of life and death and rebirth and regeneration.

Although our floristic list (Table 2) includes plant species mentioned in the Bible [57] and identified in very old artworks [2,30,58], surprisingly, some common Mediterranean plants, such as *Ficus carica* L., *Laurus nobilis* L., *Paliurus spina-christi* Mill., and *Tulipa* spp., were not found in the present study. In addition, only plant species from the Orchidaceae family can be considered native to Mediterranean small tree vegetation (maquis) or dry grassland habitats.

It is not known how much influence the regiment had on the appearance or content of the artwork. The Pelješac peninsula was a very rural area, where the inhabitants lived in poverty, and, in general, there was no need to show native plants, which people knew well anyway. However, it is important to emphasise that, for the first time, the inhabitants had the opportunity to see the appearance of the figures of Christ, the Mother of God, and the saints, which until then they had only heard about in church services [59].

Regardless of the poverty of the local population, all the artworks studied were donated by the faithful for sacred monuments and were in situ at the time of acquisition. The regiment freely commissioned the artworks for the churches, which the Church accepted. The authors of some artworks are not known, although the literature emphasises that they were under the influence of central and southern Italian artists [16]. Filippo Naldi painted almost as a rule for the poor who lived after the withdrawal of the Ottoman Turks in a wide area of what is now southern Croatia (Dalmatia), including the Pelješac peninsula [59]. His training in painting is unclear: he served in the Venetian army and was a port administrator in a small town not far from the northern coast of the Pelješac peninsula.

The floristic list offered here for the first time for the Croatian coastal region cannot be completed without an adequate base of artworks from other parts of the eastern and western Adriatic coast. However, a comparison and analysis could be made, at least in part, with the flora listed in a botanical database of ancient Roman paintings and sculptures, which includes a dataset of about 420 artworks [2]. The floristic study of Roman iconography included a large number of botanical elements (168 species, 78 families, and 159 genera) and shows some similarities to the most common plants (*Acanthus mollis*, *Vitis vinifera*) found in our study. However, a high proportion of phanerophytes and geophytes, as well as the presence of some taxonomic (e.g., pteridophytes) or functional (e.g., macrophytes) groups, were not found in our case.

Some similarities in the plant record are found in Islamic art. Although Islamic art is not art of a specific religion, time, place, or of a single medium (it spans ca. 1400 years, covers many lands and populations, and includes a range of artistic fields), the Ottomans (1299–1923) not only brought a new level of naturalism and detail to the design of flowers in Islamic artwork, especially in ornaments, but also introduced tulip and hyacinth to already developed floral motifs, such as lotus, lily, peony, chrysanthemum, and carnation [60]. In general, floral motifs in Islamic art avoid a focus on concepts of realism, such as growth or life [61]. Certain types of flowers or plants can have theological meanings; for example, the cypress often represents humility before God. From the late 16th to the mid-18th century, classical Ottoman artwork, especially in architecture, gradually lost ground to emerging western Baroque influences, and Baroque ornamentation became dominant even in famous religious buildings (e.g., Laleli Madrasa, Istanbul). In the religions of Buddhism and Hinduism, the lotus (genus *Nelumbo*) is the most commonly depicted plant and is associated with purity and beauty. Even a variety of colors are associated with different aspects of Buddhism; for example, the blue lotus flower is associated with the victory of the spirit over that of wisdom, intelligence, and knowledge [62]. In our study, the lotus flower (*Nelumbo nucifera* Gaertn.) was not found, though it was presented in Italian artworks [2].

## 5. Conclusions

A high proportion of non-native plants, mostly small trees or shrubs, from the Palaearctic and Americas was noted. The most common plant species recorded in the study are found not only in the artworks of the Baroque period, but also in artworks from various historical periods and, sometimes, from other religions. We assumed that the plants were selected for decorative and aesthetic reasons, as well as for their symbolic significance.

We believe that the most important result of this work lies in the information discovered about the botanical biodiversity of Baroque iconography in Croatia. However, considering the relatively small area and the artworks studied, the results should be read and analysed in the context of a better understanding of the cultural heritage, natural history, and knowledge of people from the Baroque period and possible higher agricultural land use in this part of the Mediterranean in the last few centuries.

## Figures and Tables

**Table 1:** List of studied artworks with codes, name, and location of church/monastery, and year (period) of its construction.

Code	Artwork	Shown on Figures
1.	St. Anne and Our Lady with Jesus Christ and Our Father [unknown Baroque painter St. Anne Chapel, Žukovac near the village of Kućište, 1625].	Figure 2A–C.
2.	The altar of St. Francis of Assisi [The Franciscan Monastery and Church of the Great Lady, Podgorje, late 15th century].	Figure 3A–C.
3.	The altar St. Anthony of Padua [ibid].	Figure 3D,E.
4.	The altar of St. Anthony of Padua [The church of Our Lady of Carmel, the village of Carmel, Podgorje, near Orebić, 1470].	Figure 4A.
5.	The altar of St. Anthony of Padua [ibid].	Figure 4C,D.
6.	The altar of St. Anthony of Padua [ibid].	Figure 4F.
7.	The altar of St Joseph [ibid].	Figure 4E.
8.	The Archangel Gabriel [ibid].	Figure 4B.
9.	The Escape to Egypt [unknown Baroque painter Church of Christian’s helpers, Orebić, 1853–1886].	Figure 2D.
10.	Health-related Votive Tablet [ibid].	Figure 2E.
11.	The antependium [painted by Filippo Naldi, mid-18th century, St. Anthony of Padua Church, Trpanj, 1695].	Figure 5A.
12.	The Virgin with Child, St. Blasius, and St. Nicholas [painted by Filippo Naldi, oil on canvas, 198 × 100 cm, mid-18th century, the parish church of St Blasius, Janjina, after 1774].	Figure 2F.
13.	The antependium from altar of Our Lady of Mercy [painted by Filippo Naldi and St. John the Baptist, mid-18th century, the parish church of St. Martin, Žuljana, 1556].	Figure 5B.
14.	The antependium from altar of St. John the Baptist [ibid].	Figure 5C.
15.	Birth and Death with the Seven Holy Sacraments [painted by Filippo Naldi, oil on canvas, 700 × 100 cm, wooden fence at the choir, mid-18th century, the parish church of Our Lady of the Rosary, Tomislavovac near the village of Putniković, 1569].	Figure 5D–F.

## Data Availability

Data are contained within the article.
